# Anaesthetic Management of A Patient with Hypokalemic Periodic Paralysis- A Case Report

**Published:** 2009-04

**Authors:** S Chitra, Grace Korula

**Affiliations:** 1 Lecturer, Department of Anesthesia, Christian Medical College, Vellore; 2 Professor, Department of Anesthesia, Christian Medical College, Vellore

**Keywords:** Hypokalemic periodic paralysis, Anaesthesia, Perioperative paralysis, Hypokalemia

## Abstract

**Summary:**

We report the anaesthetic management of a patient with hypokalemic periodic paralysis who underwent hepaticojejunostomy for stricture of the common bile duct. Patients with this disorder, who are apparently normal, can develop sudden paralysis as they are exposed to many of the predisposing factors, perioperatively. The complications due to this rare genetic disorder, the factors that can precipitate these problems and preventive measures are discussed.

## Introduction

Hypokalemic periodic paralysis is a rare genetic disorder characterised by recurrent attacks of skeletal muscle weakness with associated hypokalemia which is precipitated by stress, cold, carbohydrate load, infection etc. There is an increased risk of pre and post anaesthetic paralysis. Factors that cause a decrease in serum potassium will precipitate attacks of paralysis.^1^–^4^. The perioperative management of a patient with hypokalemic periodic paralysis is challenging in view of the increased risk of paralysis. The key to successful management of such a patient is to avoid triggering factors, vigilant perioperative monitoring of potassium and aggressive treatment of hypokalemia when it occurs.

## Case report

A 44 year old male patient, a known case of hypokalemic periodic paralysis with stricture of the common bile duct (CBD) was scheduled for hepaticojejunostomy. He had an uneventful cholecystectomy and choledocholithotomy 4 years prior to this. 5 months later he developed jaundice and fever with weakness of upper and lower extremities. On evaluation he was found to have low serum potassium. The paralysis improved after 3 days of oral potassium. A similar episode, which also responded to oral potassium, occurred 6 months before this admission. There was no respiratory muscle involvement.

On arrival to our hospital, a diagnosis of hypokalemic periodic paralysis, associated vitamin D deficiency and secondary hyperparathyroidism attributed to CBD stricture was made. He was treated with oral spironolactone 50mg once a day and calcitrol granules. He was a smoker. His effort tolerance was 2-3 kilometers on level ground. On preoperative physical examination, he was moderately built, weighing 72kg. There was no icterus. The baseline heart rate was 72/min and blood pressure 110/70mmHg. His chest was clear on auscultation. No cardiovascular abnormality was detected. He had no neurological deficit, muscle tone and power were normal. The airway assessment revealed a Mallampati class 2 with normal neck and jaw movement.

Laboratory finding were hemoglobin 12.4g/dl, serum potassium 3.5mEq/L, calcium 8mg/dl and phosphorus 2.9mg/dl. The liver function tests showed total bilirubin 1.7mg/dl, SGOT 63U/L, SGPT 121U/L and alkaline phosphatase 136U/L. Bleeding parameters were normal. Arterial blood gas analysis: pH 7.396, PCO2 40 mm Hg, PO2 67.4 mm Hg, HCO3 24.0mEq/L, BE-0.3, SaO2 93%. The parathormone levels were elevated initially. His thyroid function tests were normal. The chest X-ray, ECG and electromyography done was normal.

Oral potassium was continued till the morning of surgery and the morning serum potassium was 3.9 mEq/L. Oral diazepam 10mg was given as premedication along with metoclopramide 10mg. We planned to give a general anaesthetic supplemented with epidural. On arrival to the OR after initiating monitoring using ECG, pulse oximetry and NIBP and some further sedation with 1mg of midazolam an epidural was sited at T8-T9 level with 18 G Tuohy needle. After confirming position of the catheter, 6ml of 0.25% bupivacaine along with 50 μg of fentanyl were given in 2 increments. Anaesthesia was induced with fentanyl 100 μg, thiopentone sodium 250 mg and atracurium 40 mg. Trachea was intubated using 8.5 mm cuffed endotracheal tube. Hypocapnia was avoided using end tidal carbon dioxide monitor. The right internal jugular vein and right femoral artery were cannulated under aseptic precautions. An oropharyngeal temperature probe was used to monitor intraoperative temperature. Measures were taken to prevent hypothermia which included plastic drapes to cover the patient, hot air warming device, fluid warmer for IV fluids and HME filter for humidification of inhaled gases. Anaesthesia was maintained with isoflurane 2% in air oxygen mixture with increments of atracurium for muscle relaxation under neuromuscular monitoring. This was supplemented with epidural analgesia using 5 ml/hr of bupivacaine 0.1% and fentanyl 2μg/ml. Maintenance fluid was Ringer lactate adjusted to maintain a CVP of 8-9 mmHg. A slow infusion of 20 mEq of potassium was infused. Intraoperative potassium were 3.9 and 4.0 mEq/L. The surgery lasted 3 hours. The hemodynamics were stable throughout the procedure with a heart rate 86 to 88/min and a mean blood pressure of 60-70 mmHg. The blood loss was 300ml.

An arterial blood gas analysis done intraoperatively showed pH 7.279, PCO_2_ 46.2 mmHg, PO_2_ 134.1mmHg, HCO_3_ 21.0mEq/L, BE-4.3. The neuromuscular blockade was reversed and patient was extubated at the end of the procedure. He was shifted to High Dependency Unit for post operative monitoring. In the postoperative period, potassium was supplemented IV with daily monitoring of serum potassium.

The lowest measurment was 3.3mEq/L but there were no episodes of paralysis. The epidural catheter was left in situ for 48 hrs postoperatively with 8ml/hr of 0.1% bupivacaine and 2μg/ml fentanyl. He was discharged on the sixth postoperative day with advice to take syrup potassium and spironolactone.

## Discussion

Hypokalemic familial periodic paralysis(HOKPP) is a rare genetic disorder with autosomal dominant inheritance and male preponderance^1^,^2^. The disease is linked to chromosome 1q31-32, the region within the gene encoding for the dihyropyridine-sensitive calcium channel.^2^ The mechanism for a decrease in potassium is felt to be associated with abnormal uptake of potassium by the muscle cells which alters membrane potential and renders skeletal muscle inexcitable and not potassium loss.^3^,^4^ HOKPP is characterized by two different forms: a paralytic and a myopathic form.^5^ In the paralytic form as in our patient, recurrent attacks of reversible flaccid paralysis with a concomitant hypokalemia leading to paraparesis or tetraparesis occur, sparing the respiratory muscles.^1^ The myopathic form, a fixed muscle weakness, slowly progresses and presents as exercise intolerance predominantly of the lower limbs.

HOKPP patients are at increased risk for pre- or post-anaesthetic paralysis. Factors triggering paralytic attacks are unusually strenuous effort, excess of carbohydrate-rich meals, alcohol, infection, menstruation, pregnancy, glucose infusion, insulin, hypothermia, metabolic alkalosis, anaesthesia and steroids.^1^,^3^,^4^,^6^,^7^ Metabolic changes and medications causing a decrease in serum potassium precipitate attacks and those causing an increase may abort an attack.^7^ Guidelines for perioperative care include close control of plasma potassium concentration, avoidance of large glucose and salt loads, maintenance of body temperature, acid-base balance, and careful use of neuromuscular blocking agents.^1^,^4^,^6^,^7^ Good premedication to allay anxiety, avoidance of stress and adequate analgesia is vital in preventing an attack.

A potential link between malignant hyperthermia (MH)and HOKPP has been suggested.^8^ However there are previous reports of the use of volatile agents without harm and there has not been conclusive evidence in support of the hypothesis that MH and HOKPP are allelic.^9^ Lehmann-Horn and Iaizzo found no association with MH, but stressed that the anaesthetist should be prepared for an anaesthetic- induced reaction and suggested non-triggering anaesthesia.^10^

Our patient received a light meal the night before surgery. He received a sedative oral premedication and intravenous midazolam before siting the epidural. We chose to use general anaesthesia using isoflurane. He had already received an uneventful general anaesthesia 4 years back. The alternative was to use TIVA, but without the use of target controlled infusion pump and the use of BIS, judging the depth of anaesthesia is difficult. We avoided succinylcholine and halothane. Intraoperative epidural infusion reduced stress and atracurium dose was adjusted with neuromuscular monitoring. Non depolarizing muscle relaxants have been used for HOKPP without adverse events,^4^,^7^,^11^ but some authors recommend avoidance of neuromuscular blockade.^6^ The mechanism of the disease is not related to the neuromuscular junction; thus the magnitude of the effect of non depolarizing muscle relaxants should not differ from normal patients.^3^,^4^ Depolarising muscle relaxants should not be used because of alterations in membrane potential and alterations in electrolyte levels with the possibility of prolonged weakness.^1^ The short acting relaxants are preferable to long acting drugs.^1^,^3^,^11^

Intravenous fluids used were Ringer lactate and normal saline with potassium supplement. Glucose solutions are contraindicated and the use of normal saline should be restricted. The serum potassium values measured intraoperatively were normal. If serum potassium is low, mannitol can be used as a vehicle to supplement potassium.^12^ The temperature was kept above 36°C throughout surgery. Ventilation was adjusted to a ‘controlled hypercapnia’ (pCO2 above 40 mmHg) to avoid alkalosis. The mild acidosis found on blood gas analysis was not treated to prevent hypokalemia.

Cardiac arrhythmia can arise not only from hypokalemia but also from associated cardiac dysfunction and myopathy in these patients. ECG signs for hypokalemia is more pronounced than in normal persons with the same level of hypokalemia.^6^ Maintaining normal serum potassium will prevent arrhythmia related to hypokalemia only.

Kramer et al have reported cardiac dysfunction in a young adult who presented with HOKPP with increased myocardial fraction of CPK (myocardial band), alteration in the lactic dehydrogenase isoenzyme pattern, severe bradycardia and decreased left ventricular dysfunction.^13^ There have been other reports of arrhythmias with Thyrotoxic variety of HOKPP. This variety is more common in Asian countries and hence this should be kept in mind while evaluating patients with HOKPP in these countries.^14^

Fluctuations in electrolytes, infection and pain can lead to paralysis in the postoperative period. Epidural analgesia helped our patient have an uneventful perioperative course.^2^,^15^ Hypokalemia manifests earlier than paralysis and so correction can prevent paralysis.^3^

In conclusion we report the anaesthetic management of a case of hypokalemic periodic paralysis who underwent a major upper abdominal surgery. Adequate preoperative preparation, vigilant intraoperative monitoring of potassium and aggressive correction of hypokalemia, avoiding factors that can trigger hypokalemia and good postoperative pain relief measures go a long way in the successful management of these groups of patients.

## References

[1] Hofer C, Zalunardo MP, Zollinger A (2001). Total intravenous anesthesia in a patient with familial hypokalemic periodic paralysis. Anesthesia.

[2] Robinson JE, Morin VI, Douglas MJ, Wilson RD (2000). Familial hypokalemic periodic paralysis and Wolff Parkinson White syndrome in pregnancy. Canadian journal of anaesthesia.

[3] Stoelting RK, Diersdorf SF Anesthesia and coexisting diseases. Skin and Musculoskeletal diseases.

[4] Lema G, Urzna J, Moran S, Canessa R (1991). Successful anaesthetic management of a patient with hypokalemia familial periodic paralysis undergoing cardiac surgery. Anesthesiology.

[5] Links TP, Zwarts MJ (1990). Permanent muscle weakness in familial hypokalemic periodic paralysis clinical, radiological and pathological aspects. Brain.

[6] Melnick B, Chang J, Larson CE, Bedger R (1983). Hypokalemic familial periodic paralysis. Anesthesiology.

[7] Rollman JE, Dickson CM (1985). Anesthetic management of a patient with hypokalemic periodic paralysis for coronary artery bypass surgery. Anesthesiology.

[8] Lambert C, Blanloeil Y, Krivosic R, Berard L, Reyford H, Pinaud M (1994). Malignant hyperthermia in a patient with hypokalemic periodic paralysis. Anesthesia and Analgesia.

[9] Marchant CL, Ellis FR, Halsall PJ (2004). Mutation analysis of two patients with hypokalemic periodic paralysis and suspected malignant hyperthermia. Muscle Nerve.

[10] Lehman - Horn F, Iaizzo PA (1990). Are myotonias and periodic paralysis associated with susceptibility to malignant hyperthermia?. British Journal of Anaesthesia.

[11] Rooney RT, Shanahan EC, Sun T, Nally B (1988). Atracurium and hypokalemic familial periodic paralysis. Anesthesia and Analgesia.

[12] Griggs RC, Resnick J, Engel WK (1983). Intravenous treatment of hypokalemic periodic paralysis. Arch Neurol.

[13] Kramer LD, Cole JP, Messenger JC (1975). Cardiac dysfunction in a young adult with hypokalemic periodic paralysis. Chest.

[14] Pompeo A, Nepa A, Maddestra M (2007). Thyrotoxic hypokalemic periodic paralysis; an overlooked pathology in western countries. European Journal of Internal medicine.

[15] Viscom CM, Ptacek LJ, Dudley D (1999). Anaesthetic management of hypokalemia familial periodic paralysis during parturition. Anesthesia and Analgesia.

